# Tolerance Analysis of Test Mass Alignment Errors for Space-Based Gravitational Wave Detection

**DOI:** 10.3390/s25237393

**Published:** 2025-12-04

**Authors:** Jun Ke, Ruihong Gao, Jinghan Liu, Mengyang Zhao, Ziren Luo, Jia Shen, Peng Dong

**Affiliations:** 1 School of Fundamental Physics and Mathematical Sciences, Hangzhou Institute for Advanced Study, University of Chinese Academy of Sciences, Hangzhou 310024, China; 2 Institute of Mechanics, Chinese Academy of Sciences, Beijing 100190, China; 3 National Space Science Center, Chinese Academy of Sciences, Beijing 100190, China; 4 University of Chinese Academy of Sciences, Beijing 100049, China

**Keywords:** space-based gravitational wave detection, test mass interferometers, tilt-to-length coupling noise, tolerance analysis, optical simulation

## Abstract

Space-based gravitational wave detection imposes extremely high requirements on displacement measurement accuracy, with its core measurement components being laser interferometers and inertial sensors. The laser interferometers detect gravitational wave signals by measuring the distance between two test masses (TMs) housed within the inertial sensors. Spatial alignment errors of the TMs relative to the laser interferometers can severely degrade the interferometric performance, primarily by significantly amplifying tilt-to-length (TTL) coupling noise and reducing interferometric efficiency. This paper presents a systematic analysis of the coupling mechanisms between TM alignment errors and TTL coupling noise. We first establish a comprehensive TTL noise model that accounts for alignment errors, then verify and analyze it through optical simulations. This research ultimately clarifies the coupling mechanisms of TM alignment errors in the context of space-borne gravitational wave missions and determines the allowable alignment tolerance specifications required to meet the gravitational wave detection sensitivity requirements. This work provides critical theoretical foundations and design guidance for the ground alignment procedures and on-orbit performance prediction of future space-based gravitational wave detection missions.

## 1. Introduction

Space gravitational wave detection utilizes laser interferometry technology to capture extremely weak gravitational wave signals generated by cosmic events, such as black hole mergers, supernova explosions, etc. [1], thereby revealing the mysteries of the deep universe and verifying Einstein’s theory of general relativity [2,3]. Unlike ground-based gravitational wave detectors, such as the Laser Interferometer Gravitational-Wave Observatory (LIGO) [4] and Virgo [5], space-based detectors, such as the Laser Interferometer Space Antenna (LISA) [6] and the Taiji program [7], deploy multiple spacecraft in space to form extremely long baselines.

**Figure 1:**
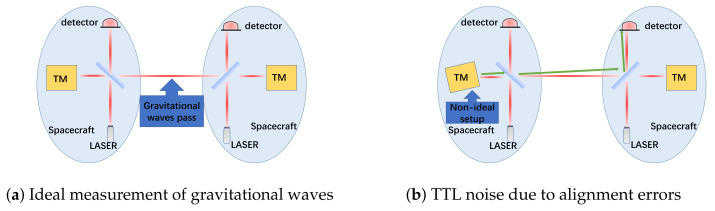
Simplified schematic of a single intersatellite laser link for space-based gravitational wave detection (the third spacecraft is omitted for clarity, and the detailed split interferometry composed of interspacecraft and TM interferometers is neglected): (**a**) Under ideal alignment conditions, gravitational waves induce genuine optical path changes through the stretching and compression of spacetime that are accurately measured; (**b**) With TM alignment errors, angular jitter produces spurious optical path variations (TTL noise) that can mask true gravitational wave signals. The green arrows indicate the altered optical path due to alignment errors.

The core measurement components of space-based gravitational wave detection are laser interferometers and inertial sensors [8,9]. The laser interferometers detect gravitational wave signals by measuring the distance between two TMs housed within the inertial sensors. These TMs serve as inertial references that ideally follow geodesic motion in spacetime, free from non-gravitational forces. The fundamental measurement principle relies on detecting minute changes in the distances between freely floating TMs caused by passing gravitational waves. As illustrated in Figure 1a, when a gravitational wave passes through the detector, it periodically stretches and compresses spacetime, inducing picometer-scale variations in the interferometer arm lengths. These distance variations, typically on the order of picometers (pm), are detected as phase shifts in the laser interferometer and serve as direct evidence of gravitational waves [10].

However, as shown in Figure 1b, in practical systems with alignment errors, even in the absence of gravitational waves, angular jitter of the TMs can couple into the longitudinal displacement measurement through TTL effects, creating noise that can obscure the genuine gravitational wave signals. This highlights the critical importance of precise TM alignment for achieving the required detection sensitivity.

The optical surface of the TM serves as the reflective interface for the laser interferometer. Variations in its position and orientation directly alter the measurement optical path, consequently affecting the final measurement accuracy [11]. As illustrated in Figure 1b, even when the distance between two TMs remains constant and no gravitational wave is present, changes in the angle between the surface normal and the incident beam can introduce additional optical path differences (OPD) through TTL coupling effects. Since the TM and interferometer must be integrated and precisely aligned on the ground, the six-degree-of-freedom alignment errors introduced during ground assembly will directly determine the performance of on-orbit measurements [12]. Therefore, a fundamental challenge in the integration of the TM interferometer is the lack of a defined methodology and specific values for alignment tolerances. How to determine the TM alignment tolerance remains an open question. This work addresses this by proposing that the tolerance must be derived from two concurrent constraints: the allowable TTL coupling noise and the required interference efficiency. The following sections will elucidate why these two factors are the key determinants for the alignment specifications.

TTL coupling noise represents one of the principal noise sources in space laser interferometers [13], originating from the coupling between angular jitter and optical path length measurements. This effect, as schematically shown in Figure 1b, occurs when the reference beam strikes the detector center perpendicularly while the measurement beam arrives at an oblique angle, causing the measurement beam to traverse an extended optical path compared to the reference beam. Consequently, TTL coupling noise is not generated when the TM rotates about the laser propagation axis, but emerges during rotations about other axes.

Beyond directly affecting TTL coupling noise, alignment errors between the TM and interferometer can also perturb the conjugate optical system, indirectly intensifying TTL coupling noise. Currently, employing conjugate optical imaging systems constitutes one of the primary methods for TTL coupling noise suppression [14,15]. These systems image the TM’s jitter rotation center onto the detector photosensitive surface, effectively suppressing TTL coupling noise under ideal conditions. However, when a displacement exists along the optical axis between the TM’s jitter rotation center and the object point, the object-image conjugate relationship is disrupted, consequently compromising the conjugate imaging system’s capability to suppress TTL coupling noise.

In interferometric measurements, a higher overlap ratio between the two laser beams yields superior interference fringe contrast, enabling the detector to receive the carrier signal more distinctly and thereby achieve more accurate phase demodulation and physical quantity measurement. The ratio of optical energy participating in interference fringe formation to the total input optical energy is defined as interference efficiency. TM alignment errors can alter the laser propagation path, modifying the overlap ratio between interfering beams, thus degrading interference efficiency.

In summary, alignment errors of the TM relative to the interferometer impact both TTL coupling noise and interference efficiency, establishing crucial criteria for determining TM tolerance ranges. As conceptually illustrated in Figure 1b, these alignment-induced effects can generate noise signals that mimic or obscure genuine gravitational wave signatures shown in Figure 1a. While substantial research has been conducted on TTL coupling noise theoretical models, these models often incorporate specific conditional assumptions and require validation through simulation and experimentation before practical application. Consequently, there is an urgent need for systematic simulation of TM alignment error effects on TTL coupling noise and interference efficiency to guide engineering practice.

Addressing this need, this paper focuses on the practical engineering requirements of space gravitational wave detection missions, systematically investigating the coupling mechanisms of six-degree-of-freedom TM alignment errors on TTL coupling noise and beam overlap performance. By establishing theoretical models and implementing joint simulation chains, we quantitatively analyze system performance under combined multi-error effects, ultimately proposing alignment tolerance ranges that satisfy the overall noise budget. This work provides critical foundations for ground alignment procedures and on-orbit performance predictions.

Section 2 introduces the TTL coupling noise model, establishing the theoretical basis for simulation design; Section 3 elaborates on the optical system design and simulation methodology, providing an overview of the workflow and methods; Section 4 presents detailed simulation processes and results following Section 3’s methodology; Section 5 summarizes findings from Section 4 and presents principal conclusions.

## 2. TTL Coupling Noise Model

TTL coupling noise originates from the coupling effect between beam angular jitter and geometric deviations in the optical system [13]. TTL coupling noise is an essential index for evaluating the performance of space gravitational wave telescope optical systems. Manufacturing errors can cause wavefront error, low-order aberration ratio, and TTL coupling noise cancellation ratio to deviate from the ideal design value, degrading the performance of telescopes [16]. This coupling induces non-common-mode variations in the optical path lengths of the two interferometer arms. Space-based gravitational wave detection imposes extremely high requirements on the relative alignment errors between the TMs and the interferometers. As mentioned in Section 1, six-degree-of-freedom alignment errors will directly couple into the interferometric measurement, introducing significant TTL noise, which is key to determining system performance and ground alignment tolerance. To systematically analyze its impact, this section establishes a geometric TTL coupling noise model.

### 2.1. Geometric TTL Coupling Noise Model

Geometric TTL coupling primarily originates from two basic physical effects: (a) lever arm effect and (b) piston effect.

**Figure 2:**
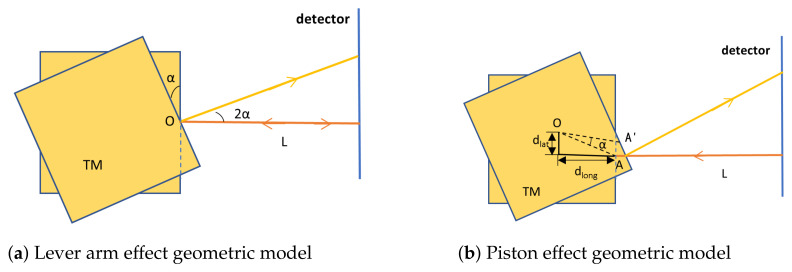
Schematic diagram of the decomposed geometric TTL coupling noise model. In the diagrams, orange lines indicate the original optical path, and yellow lines indicate the optical path after TM rotation. (**a**) Lever arm effect: The TM rotates by an angle α around the reflection point A, where the center of rotation coincides with the reflection point. The reflected beam direction deflects by 2α, causing the geometric path to the detector to lengthen. The resulting ${\mathrm{OPD}}_{\mathrm{lever}}$ is the source of pure second-order TTL coupling. (**b**) Piston effect: The TM rotates by an angle α around the center of rotation O, where offsets exist between O and the reflection point A: lateral offset $d_{\mathrm{lat}}$ and longitudinal offset $d_{\mathrm{long}}$. The reflection point moves from A to $A^{\prime }$. Its longitudinal displacement $p_{\mathrm{long}}$ causes changes in the incident and reflected optical path lengths, constituting the primary source of TTL coupling. In this effect, $d_{\mathrm{lat}}$ dominates the first-order coupling, while $d_{\mathrm{long}}$ contributes to the second-order coupling.

**(a) Lever Arm Effect** The lever arm effect describes the optical path change introduced by pure angular deflection when the TM rotates around its reflection point. Its physical model is shown in Figure 2a.

In this ideal scenario, the position of reflection point A remains fixed. When the TM rotates by an angle α around point A, according to the law of reflection, the direction of the reflected beam deflects by 2α. This deflection increases the geometric path length the beam must travel to reach the detector. As shown in Figure 2a, the resulting OPD can be calculated as [11,17]:

(1) $${\mathrm{OPD}}_{\mathrm{lever}}=\left(\frac{1}{\cos(2\alpha )}-1\right)L\approx 2L\alpha^{2}.$$

Here, *L* is the projection distance from reflection point A to the detector. Equation (1) shows that the lever arm effect contributes a pure second-order term, making it a source of second-order TTL coupling.

**(b) Piston Effect**

In practical systems, the mechanical center of rotation of the TM is typically located at its center of mass, which does not coincide with the optical reflection point, leading to the piston effect. Its physical model is shown in Figure 2b.

When the TM rotates by an angle α around the center of rotation O, the reflection point moves from A to $A^{\prime }$. Based on geometric rotation relationships, the longitudinal displacement of the reflection point, denoted as $p_{\mathrm{long}}$, is the direct source of the optical path change. Its expression is given by [17]:

(2) $$p_{\mathrm{long}}=d_{\mathrm{lat}}\sin\alpha +d_{\mathrm{long}}\left(1-\cos\alpha \right).$$

This displacement introduces an OPD. Under small-angle approximation, the OPD introduced by the piston effect can be expressed as:

(3) $${\mathrm{OPD}}_{\mathrm{piston}}\approx 2d_{\mathrm{lat}}\alpha +d_{\mathrm{long}}\alpha^{2}.$$

When both lever arm and piston effects are present, the total geometric TTL coupling noise can be approximated by the linear superposition of their contributions. The TTL coupling coefficient, defined as the sensitivity of the OPD to angular jitter, is $k_{\Delta s-\alpha }=\partial \left(\mathrm{OPD}\right)/\partial \alpha$. This model clarifies the impact of various alignment error parameters: first-order TTL coupling is entirely determined by the lateral offset $d_{\mathrm{lat}}$, while second-order TTL coupling is jointly contributed by $d_{\mathrm{long}}$ and *L*. This theoretical analysis provides the key basis and optimization direction for the simulation design and tolerance analysis in Section 3 and Section 4.

### 2.2. TTL Coupling Suppression Based on Conjugate Imaging System

The preceding analysis indicates that geometric TTL coupling, particularly the first-order term dominated by $d_{\mathrm{lat}}$ and the second-order terms contributed by $d_{\mathrm{long}}$ and *L*, is a key factor limiting measurement accuracy. Various methods have been developed to suppress TTL coupling noise [14,18,19], among which the implementation of conjugate imaging systems has emerged as a particularly effective and widely adopted approach in current practice [14,15].

**Figure 3:**
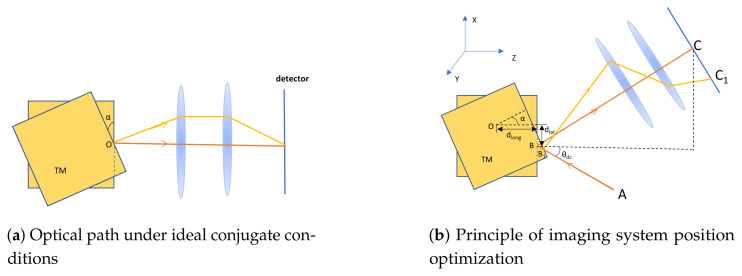
Schematic diagram of the TTL suppression principle using a conjugate imaging system. (**a**) Ideal conjugate condition: The TM’s rotation center (object point O) is strictly conjugated to the detector surface (image point) via the imaging system. When the TM rotates around the object point O by α, according to Fermat’s principle, all optical paths from point O to the image point via the imaging system have equal optical path lengths. Consequently, angular jitter of the reflected beam does not introduce optical path changes, and the lever arm effect is completely suppressed. (**b**) Practical system optimization: When a longitudinal offset $d_{\mathrm{long}}$ exists between the TM rotation center O and the desired optical measurement point (intended object point), the ideal conjugation is disrupted. By precisely adjusting the axial position of the imaging lens group (changing parameter *k*), the second-order optical path variation introduced by $d_{\mathrm{long}}$ can be compensated, thereby effectively suppressing the corresponding second-order TTL coupling.

**(a) Ideal Conjugation and Fermat’s Principle** The core of the conjugate imaging system lies in establishing a strict object-image conjugate relationship between the jitter rotation center of the TM (object plane) and the photosensitive surface of the detector (image plane). Its basic principle is shown in Figure 3a.

When the TM rotates around the incidence point (i.e., the object point) as shown in Figure 3a, according to Fermat’s principle, all optical paths from the object point to the image point via the imaging system have equal lengths [20]. This means that even if the TM experiences angular jitter α, the optical path length remains unchanged. Therefore, under ideal conjugate conditions, the TTL coupling introduced by the lever arm effect and the beam deflection itself can be completely suppressed.

**(b) Practical System Optimization and Second-Order Term Compensation**

In practical systems, the mechanical rotation center of the TM often has a offset from the optical measurement point (object point), and initial alignment errors exist, disrupting the ideal conjugate relationship. In this case, the total OPD after introducing the imaging system can be expressed as [14]:

(4) $${\mathrm{OPD}}_{\mathrm{ima}}\approx 2d_{\mathrm{lat}}\alpha +\frac{f_{1}}{f_{2}}\left(\frac{f_{2}}{f_{1}}d_{\mathrm{long}}+k\right)\alpha^{2}.$$

Here, $f_{1}$ and $f_{2}$ are the focal lengths of the imaging lens group, and *k* is a parameter related to the axial position of the imaging lens group.

Equation (4) reveals a key characteristic: the first-order term is entirely determined by the lateral offset $d_{\mathrm{lat}}$ and the initial tilt angle $\theta_{\mathrm{dc}}$ and is not affected by the imaging system position; whereas the coefficient of the second-order term strongly depends on the lens group position parameter *k*. This characteristic enables active suppression of TTL coupling, as illustrated in Figure 3b.

By precisely adjusting the axial position of the imaging lens group in the optical path (i.e., optimizing parameter *k*), the second-order term coefficient in Equation (4) can be set to zero:

$$\frac{f_{2}}{f_{1}}d_{\mathrm{long}}+k\approx 0.$$

In this optimized state, the system can significantly suppress the second-order TTL coupling contributed by the longitudinal offset $d_{\mathrm{long}}$ and the residual lever arm effect.

### 2.3. Comprehensive Discussion on Alignment Error Parameters and Theoretical Guidance

Consider the six-degree-of-freedom errors of the TM. To precisely define these degrees of freedom and their relation to the TTL coupling model, a coordinate system is established as conceptually illustrated in Figure 3b. The optical axis direction is defined as the Z-axis. The deviations thus include three translations (ΔX, ΔY, ΔZ) and three rotations ($\theta_{X}$, $\theta_{Y}$, $\theta_{Z}$) about these axes. Among them, rotation about the Z-axis $\theta_{Z}$ is a pure rotation around the optical axis and does not change the beam incidence angle, thus it does not introduce geometric TTL coupling. The model established in this chapter provides a clear theoretical framework for analyzing the relationship between these six-degree-of-freedom alignment errors and TTL coupling noise. The influence of each degree-of-freedom parameter can be summarized as follows: The lateral translations (ΔX, ΔY) combine to form the lateral offset $d_{\mathrm{lat}}$, which is the dominant factor for first-order TTL coupling and has the most stringent tolerance requirements. The lateral rotations ($\theta_{X}$, $\theta_{Y}$) combine to form the initial tilt angle $\theta_{\mathrm{dc}}$, which simultaneously modulates the coefficients of both first-order and second-order TTL coupling, is a major source of system nonlinearity, and also requires strict control. The longitudinal translation (ΔZ) primarily affects the absolute position of the TM along the optical axis. It indirectly affects the system’s optimization state by altering the actual conjugate distance between the TM reflective surface and the imaging system, but contributes little to the longitudinal offset $d_{\mathrm{long}}$ (a relatively small internal parameter determined by mechanical structure) between the center of rotation and the reflection point. The longitudinal rotation ($\theta_{Z}$), as mentioned earlier, rotation around the optical axis does not change the beam incidence angle and thus does not introduce geometric TTL coupling; its tolerance requirements can be significantly relaxed.

The longitudinal offset $d_{\mathrm{long}}$ is a key internal geometric parameter describing the relative positional deviation along the optical axis between the TM’s mechanical center of rotation and its optical reflection point. For a cubic TM, this offset is approximately equal to half the side length, i.e., the radius of the TM. It differs in physical meaning and impact from the absolute alignment error ΔZ, which describes the overall position of the TM. $d_{\mathrm{long}}$ is a core variable directly involved in the TTL coupling calculation in the model, whereas ΔZ primarily affects system performance by perturbing the established conjugate relationship.

This theoretical analysis provides two key guidance points for the subsequent simulation and tolerance analysis work: First, it provides the theoretical criterion for determining the optimal position of the conjugate imaging system in the optical path. As shown in Equation (4), the influence of $d_{\mathrm{long}}$ can be compensated by adjusting the lens group position (*k*). This optimization process will be detailed in Section 3.4 via simulation. Second, and more importantly, it reveals that introducing an optimized conjugate imaging system fundamentally alters the landscape of system tolerance constraints. The system can effectively suppress the second-order TTL coupling associated with $d_{\mathrm{long}}$ and *L*, meaning that the alignment accuracy requirements for these parameters can be appropriately relaxed while still meeting the overall noise budget. Conversely, stricter tolerance control must be imposed on the first-order coupling dominated by $d_{\mathrm{lat}}$ and $\theta_{\mathrm{dc}}$, which the imaging system cannot suppress.

Therefore, all quantitative analysis regarding alignment tolerance in Section 4 of this paper is conducted under the condition that an optimized conjugate imaging system is integrated. This premise is core to achieving practical and feasible alignment specifications that meet engineering requirements. This theoretical model lays a solid foundation for this series of analyses.

## 3. Simulation Design and Analysis

To systematically analyze the impact of alignment errors on interferometric measurement system performance, this study establishes a comprehensive optical-numerical joint simulation framework. This section first defines the alignment error metrics for the TM in space gravitational wave detection, then details the simulation workflow and analytical methodology, followed by a description of the optical simulation model. Finally, it presents the baseline TTL coupling coefficient and interference efficiency obtained under ideal alignment conditions.

### 3.1. Constraints for Determining Alignment Tolerances

The relative displacement of laser beams on the detector surface is a critical factor affecting interference efficiency and requires stringent constraints. Beam walk refers to the lateral displacement of a beam relative to the ideal position of optical components (such as lenses, beam splitters, and detectors) during propagation through the interferometer optical path [21].

To ensure adequate interference efficiency, the overlapping area of the two interfering beams on the detector surface must exceed 90%. Through mathematical calculation, for a Gaussian beam with a waist radius of 1 mm passing through the designed imaging system, the beam radius after contraction is approximately 0.285 mm. Given a detector radius of 0.6 mm and a contracted beam radius of 0.285 mm, the maximum allowable beam walk satisfying this overlap requirement is 47 μm. This value is established as the tolerance metric for beam walk.

In space gravitational wave detection, within the laser jitter angle range of less than 400 μrad, the TTL coupling coefficient typically must not exceed 25 μm rad^−1^ [13,20,22]. In subsequent tolerance analyses incorporating alignment errors, all operational conditions must simultaneously satisfy the dual constraints of TTL coupling coefficient ≤ 25 μm rad ^−1^ within the 400 μrad laser jitter range and beam walk ≤ 47 μm.

### 3.2. Simulation Methodology

**Figure 4:**
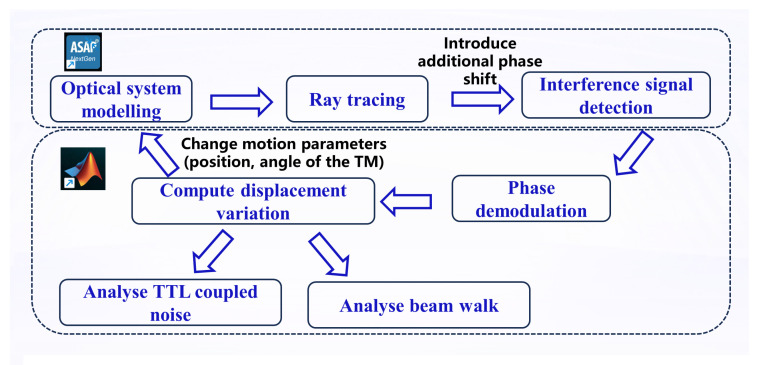
Simulation workflow for tolerance analysis of the TM interferometer. The process integrates optical simulation in ASAP with signal processing in MATLAB to extract TTL coupling coefficients and beam walk metrics.

To precisely quantify the effects of alignment errors on TTL coupling noise and interference efficiency, this study employs a systematic simulation analysis workflow that integrates the professional optical software ASAP 2019 with the numerical computing environment MATLAB 2018. The specific procedure is as follows (see Figure 4):

First, based on the optical path parameters of the Taiji program TM interferometer [23], a high-fidelity optical system model is constructed in ASAP. The model includes all critical components: light sources, mirrors, beam splitters, polarizing beam splitters, quarter-wave plates, the TM, imaging systems, and detectors.

Ray tracing is then performed in ASAP to calculate the light intensity distribution and interference field information at the detector, precisely acquiring the interference signal received by the detector. Since ASAP simulations do not include time-dependent variables and can only perform homodyne interference measurements for specific motion parameters, additional phase shifts are introduced into one beam to approximate the heterodyne interference process. To simulate the heterodyne interference process, additional phase shifts were introduced to emulate the laser’s phase evolution over one complete cycle. The phase was varied through 16 equally spaced steps covering a 2π range, thereby generating the sinusoidal interference signal for subsequent phase demodulation.

The interference signal output from ASAP is then imported into MATLAB and processed using a quadrature demodulation algorithm [24]. This algorithm discretizes the beat signal and performs orthogonal phase-locked integration over a complete 2π period to accurately extract the wrapped phase information ϕ(t).

The optical path variation ΔL is calculated from the demodulated phase change Δϕ using the fundamental principle of laser interferometry: ΔL=(λ/4π)·Δϕ, where λ is the laser wavelength. For each specified alignment error condition, the corresponding beam jitter angle α and optical path variation ΔL are computed. The TTL coupling coefficient $k_{\Delta s-\alpha }$ for that condition is then obtained by numerically differentiating ΔL with respect to α.

Finally, the relative displacement of the interference beam spots on the detector photosensitive surface, i.e., the beam walk, is calculated to serve as the key metric for evaluating interference efficiency. This integrated simulation approach leverages ASAP’s strengths in high-precision optical modeling alongside MATLAB’s powerful capabilities in complex signal processing and data analysis, ensuring the accuracy and reliability of the analytical results.

It is important to note that the numerical simulations performed in ASAP account for both geometrical and non-geometrical TTL coupling. The physical optics propagation model inherently includes wavefront distortions and diffraction effects, providing a comprehensive analysis of the TTL noise under realistic conditions. Therefore, the results presented in Section 4 already incorporate the combined impact of both coupling mechanisms.

### 3.3. Optical Model Construction

**Figure 5:**
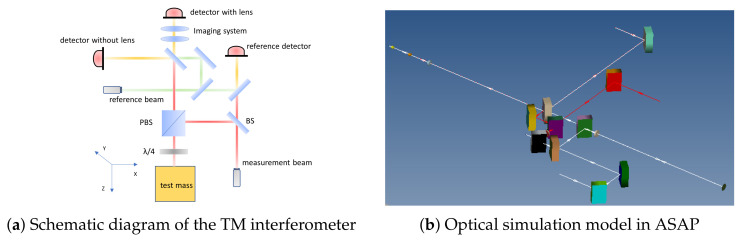
Optical model of the TM interferometer: (**a**) Schematic diagram (Red rays: measurement beam; Green rays: reference beam; Detector with lens: interference through imaging system; Detector without lens: interference without imaging system; BS: beam splitter; PBS: polarizing beam splitter; λ/4: quarter wave plate. The coordinate system (X, Y, Z) used in the analysis is indicated); (**b**) White lines represent the measurement beam and red lines represent the reference beam.

Based on the actual optical path layout and engineering parameters of the Taiji program TM interferometer [23], a high-fidelity optical simulation model was established in ASAP, as shown in Figure 5. The model comprises the following core components:

The light source system configures both measurement and reference beams as Gaussian beams with a waist radius of 1 mm, wavelength of 1064 nm, and waist locations positioned at the source. In the transmission path, the measurement beam sequentially passes through a mirror (M), beam splitter (BS), polarizing beam splitter (PBS), and quarter-wave plate before incidenting on the TM surface. After reflection, the measurement beam splits into two paths: one directly interferes with the reference beam (without passing through the imaging system), while the other passes through the designed imaging system before interfering with the reference beam.

**Figure 6:**
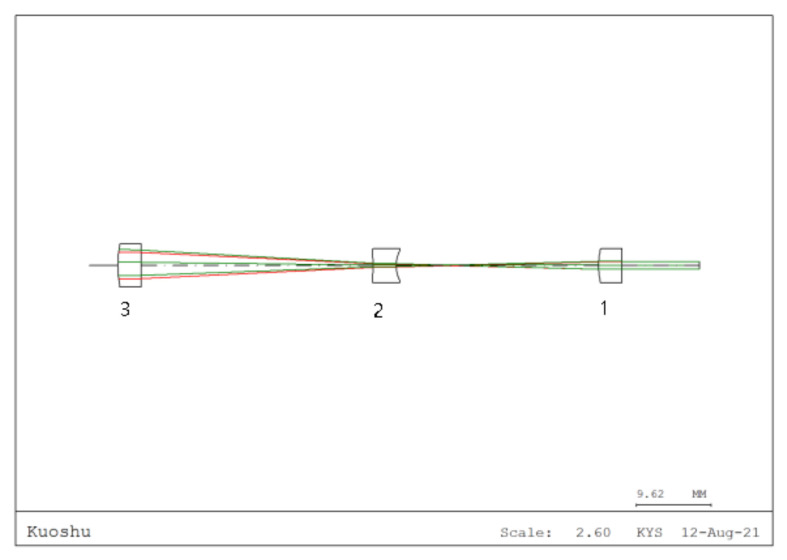
Optical design layout of the imaging system (Half field angle: $0.05^{\circ }$, beam expansion ratio: 3.5:1@1064 nm, conjugate distance: 587.2 mm. Lens 1 curvature radius: 18.81 mm, thickness: 3 mm; Lens 2 curvature radius: −36 mm, thickness: 3 mm; Lens 3 curvature radius: 9.99 mm, thickness: 3 mm).

The imaging system imports the conjugate imaging lens group optimized for the Taiji program using the CODE V 11.0,a professional optical design software, with its design layout shown in Figure 6, ensuring precise implementation of the object-image conjugate relationship. A detector with a radius of 0.6 mm is configured to receive both the “through imaging system” and “direct” interference signals for comparative analysis.

The TM is modeled as a 46 mm cube with its rotation center set at the geometric centroid (23 mm from the reflective surface), and the angular jitter range considered in the simulation is 200 μrad. To comprehensively analyze alignment error effects, the model supports parametric scanning of key parameters, including lateral offset $d_{\mathrm{lat}}$, TM longitudinal move $Z_{\mathrm{TM}}$, and initial tilt angle $\theta_{\mathrm{dc}}$. The coordinate system and rotation directions are defined as follows: the optical axis is along the Z-direction, the X-axis is perpendicular to the optical axis in the horizontal plane, and the Y-axis completes the right-handed coordinate system. The positive rotation direction follows the right-hand rule: for rotations around the X-axis, a positive angle corresponds to counterclockwise rotation when viewing from the positive X-axis toward the origin; for rotations around the Y-axis, a positive angle corresponds to counterclockwise rotation when viewing from the positive Y-axis toward the origin; for rotations around the Z-axis, a positive angle corresponds to counterclockwise rotation when viewing from the positive Z-axis toward the origin. This convention applies to both the initial tilt angle $\theta_{\mathrm{dc}}$ and the angular jitter α. Positive directions for translational offsets are defined as: positive $d_{\mathrm{lat}}$ along the positive X and Y axes, and positive $d_{\mathrm{long}}$ along the positive Z-axis (direction of beam propagation).

### 3.4. Analysis of TTL Coupling Coefficient and Beam Walk Without Alignment Errors

Under ideal conditions without alignment errors, the optimal axial position of the conjugate imaging system within the optical path was first determined. Starting from the initial position where the TM rotation center and detector surface were set in an ideal conjugate relationship, the lens group was finely translated along the optical axis in 0.1 mm increments. For each lens group position, the interference signal was acquired via ASAP simulation and processed using MATLAB to obtain the Light Path Signal Averaged Phase (LPS^AP^), thereby analyzing the TTL coupling coefficient.

The LPS^AP^ signal is obtained by averaging the LPS from each quadrant of the quadrant detector. The TTL coupling coefficient is defined as the derivative of the LPS^AP^ signal with respect to the angular jitter.

Simulations revealed that due to the introduction of optical elements such as beam splitters and wave plates, combined with the dimensional effects of the TM itself, the measurement beam could not strictly incident on the TM’s rotation center, causing the actual object-image conjugate relationship to deviate from the theoretical design. Through optimization search, the lens group position minimizing the TTL coefficient was identified as: translated 2.1 mm toward the detector direction (i.e., the last surface of the lens group positioned 5.4 mm from the detector).

**Figure 7:**
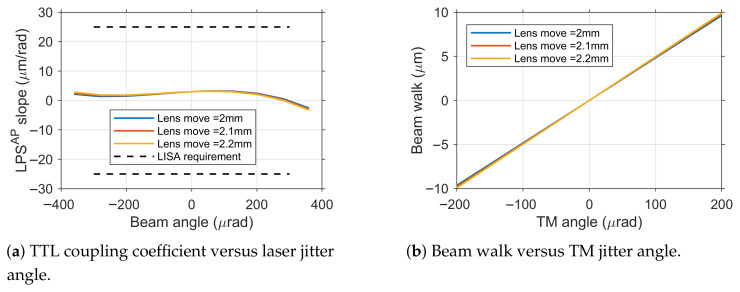
Performance with the imaging system at the optimal position and no alignment errors.

At this optimal error-free position, system performance is shown in Figure 7. Figure 7a shows the TTL coupling coefficient derived from the derivative of the LPS^AP^ signal, with a maximum value of only 3.19 μm rad^−1^, significantly lower than the LISA requirement of 25 μm rad^−1^. This demonstrates the excellent suppression capability of the conjugate imaging system against TTL coupling noise under ideal conditions. Concurrently, the variation in beam walk with TM jitter angle is shown in Figure 7b, with its maximum value well below the 47 μm tolerance limit, fully satisfying system requirements for interference efficiency. This error-free baseline condition provides an important performance reference for subsequent evaluation of alignment error impacts.

## 4. Results and Discussion

Based on the simulation model and analysis process established in Section 3, this section systematically investigates the impact of alignment errors on system performance. We first conduct a single alignment tolerance analysis by introducing individual error types under ideal conditions to determine their respective tolerance limits. Subsequently, we examine parameter coupling effects and comprehensive tolerance determination by incorporating multiple error types to analyze their interactions. Finally, based on the analytical results and considering practical alignment error levels in engineering applications, we derive the final comprehensive alignment specifications.

### 4.1. Individual Parameter Tolerance Analysis

To determine the engineering alignment tolerances for the Taiji program TM interferometer, we systematically analyzed the individual effects of key error sources on TTL coupling noise and beam walk. Based on the geometric TTL theoretical model and joint simulation results, we established the single tolerance ranges for three core parameters–lateral offset $d_{\mathrm{lat}}$, initial tilt angle deviation $\theta_{\mathrm{dc}}$, and TM longitudinal move ΔZ–that satisfy both the 25 μm rad^−1^ noise budget and beam walk requirements.

In the following single-parameter tolerance analysis, both the initial tilt angle $\theta_{\mathrm{dc}}$ and the jitter α are consistently defined as rotations around the same axis (either X or Y) for each parameter variation.

**(a) Lateral Offset Alignment Tolerance Analysis**

**Figure 8:**
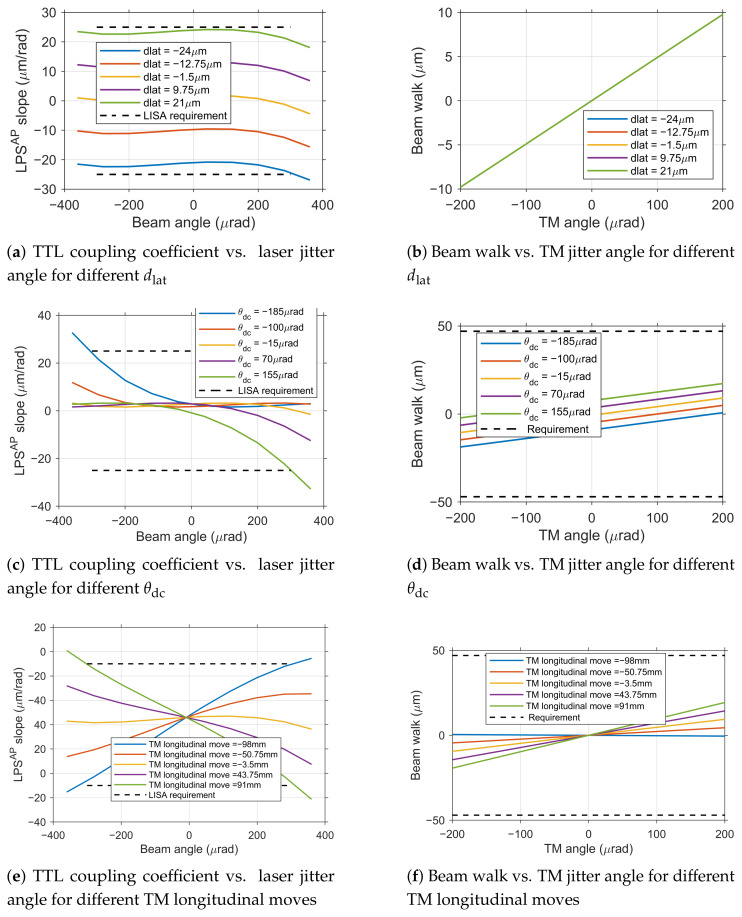
Effects of individual alignment tolerances on system performance.

Lateral offset $d_{\mathrm{lat}}$ is the primary factor contributing to first-order TTL coupling noise. As shown in Figure 8a, the TTL coupling coefficient $k_{\Delta s-\alpha }$ exhibits a significant linear relationship with $d_{\mathrm{lat}}$, validating the dominant role of the first-order term in the geometric TTL model. To simultaneously meet the dual requirements of TTL coupling noise ≤ 25 μm rad^−1^ and beam walk ≤ 47 μm, the $d_{\mathrm{lat}}$ deviation must be strictly constrained between −24 μm and 21 μm. Within this range, beam walk is minimally affected by $d_{\mathrm{lat}}$ variations (Figure 8b), with maximum displacement well below the 47 μm tolerance limit, indicating that this tolerance constraint is primarily determined by the TTL coupling noise requirement.

**(b) Initial Tilt Angle Deviation Tolerance Analysis**

As $\theta_{\mathrm{dc}}$ increases, the TTL coupling coefficient curve undergoes an overall shift (Figure 8c), while the slope of its linear segment remains relatively stable. The unilateral tolerance for $\theta_{\mathrm{dc}}$ that satisfies both noise and beam walk requirements ranges from −185 μrad to 155 μrad. Beam walk shows a good linear relationship with $\theta_{\mathrm{dc}}$ (Figure 8d), with maximum displacement remaining within safe limits.

**(c) TM longitudinal move Tolerance Analysis**

TM displacement along the optical axis ΔZ disrupts the established object-image conjugate relationship of the imaging system. As shown in Figure 8e, the system exhibits extremely high tolerance to ΔZ, with an allowable unilateral tolerance range from −98 mm to 91 mm, significantly wider than the tolerance requirements for other parameters. Beam walk is also minimally affected by ΔZ (Figure 8f).

The observed asymmetry in the tolerance ranges arises from the inherent optical asymmetries of the practical imaging lens group, which is a real design rather than an ideal thin lens.

Considering that the TM may experience simultaneous rotations around both the X and Y axes during actual operation, the TTL coupling noise power can be approximately treated as the sum of noise powers in two orthogonal directions. To ensure the total noise does not exceed the budget, the TTL coupling coefficient requirement for each direction must be divided by $\sqrt{2}$ based on the one-dimensional specification, i.e., requiring $k_{\Delta s-\alpha }<25/\sqrt{2}\approx 17.7$ μm rad^−1^. Similarly, the beam walk specification is also tightened to $47/\sqrt{2}\approx 33.2$ μm.

Under these stricter conditions, the single tolerance of each parameter is re-evaluated: the tolerance range for $d_{\mathrm{lat}}$ is tightened to −17 μm to 14 μm, the tolerance range for $\theta_{\mathrm{dc}}$ becomes −155 μrad to 125 μrad, while ΔZ still maintains extremely wide tolerance.

The single tolerance analysis results indicate that among the three key alignment parameters, lateral offset $d_{\mathrm{lat}}$ is the most critical factor constraining TTL coupling noise performance, with the most stringent tolerance requirement. Initial tilt angle $\theta_{\mathrm{dc}}$ is the next most critical, while TM longitudinal move has extremely wide tolerance.

### 4.2. Parameter Coupling Effects and Comprehensive Tolerance Determination

In practical engineering environments, multiple alignment errors typically coexist. To accurately assess the coupling effects between parameters and their comprehensive impact on system performance, this study conducted systematic simulation analyses of multiple parameter combinations, aiming to determine alignment tolerances under conditions closest to real working scenarios.

**Figure 9:**
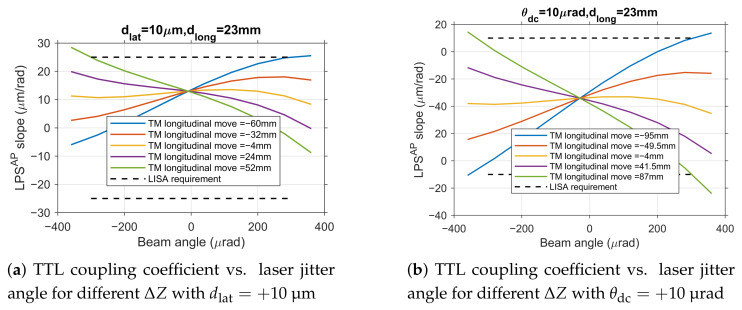

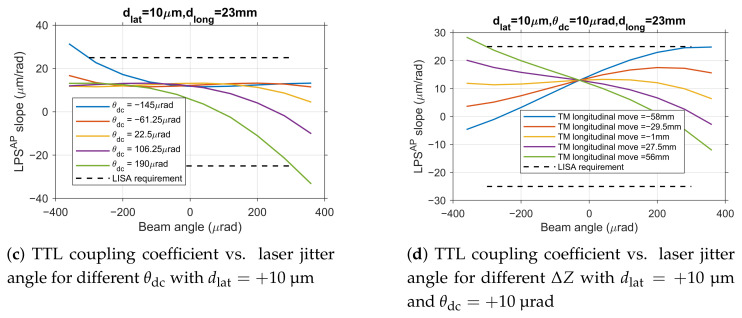
Analysis of dual-parameter coupling effects.

Simulation results indicate that the coupling effects among $d_{\mathrm{lat}}$, $\theta_{\mathrm{dc}}$, and ΔZ are weak. To quantitatively verify this, the combined impact observed in dual-parameter simulations (Figure 9) was compared against predictions derived from a simple linear superposition of the individual parameter effects characterized in Section 4.1 (Figure 8).

For instance, the TTL coupling coefficient curve for the condition $d_{\mathrm{lat}}=+10\;\mu m$ (Figure 9a) can be closely approximated by adding a constant offset (determined from the $d_{\mathrm{lat}}=+10\;\mu m$ case in Figure 8a) to the family of curves for different ΔZ under zero lateral offset (Figure 8e). Similarly, the curve for $\theta_{\mathrm{dc}}=+10\;\mu \mathrm{rad}$ (Figure 9b) aligns well with the sum of the offset from the $\theta_{\mathrm{dc}}=+10\;\mu \mathrm{rad}$ case (Figure 8c) and the baseline ΔZ curves (Figure 8e). This strong agreement confirms that their combined impact can be accurately treated as a linear superposition of individual effects. This finding significantly simplifies the complexity of tolerance allocation.

Based on engineering alignment experience, the current alignment accuracy for $d_{\mathrm{lat}}$ is approximately ±10μm. As shown in Figure 9a, under the condition of fixed $d_{\mathrm{lat}}=+10\;\mu m$ (representative of a positive deviation within the typical range), the TTL coupling coefficient curve undergoes an overall shift compared to the no-deviation state, but its trend with ΔZ variation remains essentially consistent with the single-parameter analysis, with no significant nonlinear coupling effects observed. Under these conditions, the tolerance range for ΔZ narrows from the wide single-parameter range to approximately −60 mm to 52 mm. Analysis for a negative lateral offset ($d_{\mathrm{lat}}=-10\;\mu m$) yields a symmetrically shifted curve, confirming the linear behavior for both deviation polarities.

Similarly, based on engineering alignment experience, the current alignment accuracy for $\theta_{\mathrm{dc}}$ is approximately ±10μrad. Fixing $\theta_{\mathrm{dc}}=+10\;\mu \mathrm{rad}$ (again, a representative positive value) primarily causes an offset in the zero point of the TTL coupling coefficient curve (Figure 9b), but the curve shape and its variation pattern with ΔZ remain unchanged. The tolerance range for ΔZ under these conditions only slightly narrows to −95 mm to 87 mm. The effect of a negative angular deviation is similarly linear and symmetric.

Examining the combined effect of $d_{\mathrm{lat}}$ and $\theta_{\mathrm{dc}}$, when $d_{\mathrm{lat}}$ is fixed at +10 μm, the tolerance range for $\theta_{\mathrm{dc}}$ becomes −145 μrad to 190 μrad. The variation in TTL coupling coefficient (Figure 9c) closely matches the linear sum of the individual effects from Figure 8a ($d_{\mathrm{lat}}=+10\;\mu m$) and Figure 8c (different $\theta_{\mathrm{dc}}$), further validating the non-coupling characteristics. This linearity holds for combinations involving negative deviations as well.

Based on the conclusion that parameter effects can be linearly superimposed, we further analyzed the comprehensive tolerance required to ensure system performance under conditions where multiple errors coexist, which is closest to engineering reality. Considering the presence of initial deviations of approximately ±10 μm for $d_{\mathrm{lat}}$ and ±10 μrad for $\theta_{\mathrm{dc}}$, the tolerance range for TM longitudinal move ΔZ is constrained within −70 mm to 60 mm (Figure 9d, for the case of $d_{\mathrm{lat}}=+10\;\mu m$ and $\theta_{\mathrm{dc}}=+10\;\mu \mathrm{rad}$).

The comprehensive analysis quantitatively confirms the weak coupling among the three key alignment parameters, with their combined effects being well approximated by linear superposition. This important finding provides a theoretical foundation for independent tolerance control in engineering practice, as the performance impact of any parameter combination can be reliably predicted from individual characterizations.

### 4.3. Tolerance Optimization Analysis Based on Engineering Practice

Based on the aforementioned findings, namely that TM longitudinal move ΔZ possesses both the most lenient tolerance limits and is easily controllable with precision, this study proposes a tolerance optimization scheme for engineering practice: strictly controlling ΔZ within the range of ±100 μm, and subsequently re-evaluating the tolerance limits for lateral offset ($d_{\mathrm{lat}}$) and angular deviation ($\theta_{\mathrm{dc}}$).

**Figure 10:**
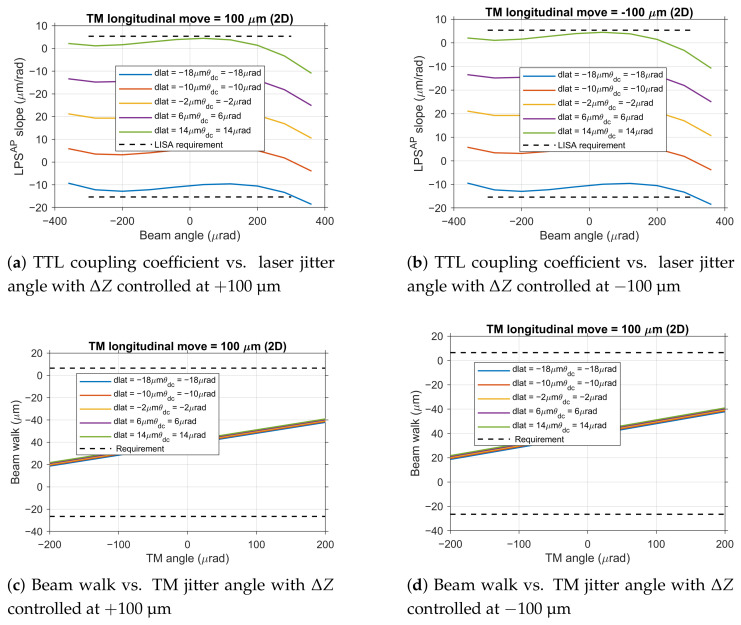
System performance analysis under engineering practice conditions.

As shown in Figure 10, simulation results demonstrate that when ΔZ is precisely controlled within ±100 μm, the system’s tolerance ranges for other alignment errors are: $d_{\mathrm{lat}}$ tolerance range of −18 μm to 14 μm, and $\theta_{\mathrm{dc}}$ tolerance range of −18 μrad to 14 μrad. This implies that the sum of translational errors of the TM in the x and y directions should be within −18 μm to 14 μm, and the sum of rotational errors around the x and y axes should be within −18 μrad to 14 μrad.

Based on the relationship between the three key parameters and the six degrees of freedom, these tolerance ranges can be translated into specific requirements for each degree of freedom as shown in Table 1.

This optimization scheme has been validated under two-dimensional rotation conditions. As shown in Figure 10, under more realistic working conditions considering TM rotation around both axes, although the TTL coupling coefficient and beam walk requirements are tightened to 17.7 μm rad^−1^ and 33.2 μm, respectively, the aforementioned tolerance ranges remain effective, demonstrating the robustness of the proposed scheme.

This optimization scheme holds significant value for engineering practice: by prioritizing control of the most easily achievable parameter (ΔZ), it provides ample tolerance space for alignment errors that are more difficult to avoid ($d_{\mathrm{lat}}$, $\theta_{\mathrm{dc}}$). Considering the linear superposition characteristics of parameter effects, in actual alignment processes, it is only necessary to control each parameter within its respective tolerance zone to ensure that the final performance simultaneously meets both the TTL coupling noise and beam walk requirements.

## 5. Conclusions

This paper addresses the practical issue of how to determine the alignment tolerance for the TM in gravitational wave interferometers. We have proposed that the tolerance should be defined by simultaneously considering the impact on TTL coupling noise and interference efficiency. Through systematic simulation based on this principle, we established the specific alignment tolerance ranges for the Taiji program, providing the essential standards for its ground integration. By establishing theoretical models and conducting joint simulations using optical software, the study determined alignment tolerance ranges that satisfy the system noise budget. Simulation results indicate that lateral offset and initial tilt angle are the key factors affecting TTL coupling noise, with the most stringent tolerance requirements, while TM longitudinal move has ample tolerance margins. More importantly, the coupling effects between parameters are weak, and their combined impact can be approximated as linear superposition, providing a theoretical basis for independently controlling various tolerances.

Based on these characteristics and comprehensive consideration of alignment levels in engineering practice, this study proposed an optimized tolerance allocation scheme: by prioritizing the restriction of easily controllable TM longitudinal move within ±100 μm, the requirements for lateral and angular deviations can be effectively relaxed. The final engineering alignment tolerances determined are: the sum of TM translational errors in the X and Y directions ($d_{\mathrm{lat}}$) must be between −18 μm and 14 μm, and the sum of rotational errors around the X and Y axes ($\theta_{\mathrm{dc}}$) must be between −18 μm and 14 μm. This scheme clarifies the critical tolerances for ground alignment and provides directly implementable technical basis for the high-precision integration of the Taiji program interferometer.

This solution successfully bridges the gap between ideal optical design and engineering practice, providing clear, quantitative, and actionable alignment guidance for the final assembly and integration of the Taiji program TM interferometer. Based on these research results, the engineering team can establish reasonable alignment process priorities: first ensure that TM longitudinal move is controlled within ±100 μm, then appropriately relax alignment requirements for lateral offset and angular deviation, thereby effectively reducing alignment difficulty and improving mission implementation feasibility, providing crucial technical assurance for the successful implementation of space gravitational wave detection missions.

## Figures and Tables

**Table 1:** Tolerance requirements for the six degrees of freedom of TM alignment.

Degree of Freedom	Tolerance Requirement
Translational degrees of freedom:	
X-direction translation (ΔX)	Must be controlled within −18 μm to 14 μm
Y-direction translation (ΔY)	Must be controlled within −18 μm to 14 μm
Z-direction translation (ΔZ)	Must be strictly controlled within ±100 μm
Rotational degrees of freedom:	
Rotation about X-axis ($\theta_{X}$)	Must be controlled within −18 μrad to 14 μrad
Rotation about Y-axis ($\theta_{Y}$)	Must be controlled within −18 μrad to 14 μrad
Rotation about Z-axis ($\theta_{Z}$)	As established in Section 2.1, rotation about
	the optical axis does not introduce TTL coupling
	noise and therefore has relaxed tolerance requirements.

## Data Availability

The original contributions presented in this study are included in the article. Further inquiries can be directed to the corresponding authors.

## Abbreviations

The following abbreviations are used in this manuscript:

TM	Test Mass
TTL	Tilt-to-Length
OPD	Optical Path Difference
LPS	Light Path Signal
AP	Averaged Phase
BS	Beam Splitter
PBS	Polarizing Beam Splitter
LISA	Laser Interferometer Space Antenna

## References

[1] Kataki A. (2024). The Role of Gravitational Waves in Understanding the Cosmic Evolution and The Underlying Physics of the Universe. Acceleron Aerosp. J..

[2] Krishnendu N., Ohme F. (2021). Testing general relativity with gravitational waves: An overview. Universe.

[3] Arun K., Pai A. (2013). Tests of general relativity and alternative theories of gravity using gravitational wave observations. Int. J. Mod. Phys..

[4] Cahillane C., Mansell G. (2022). Review of the advanced LIGO gravitational wave observatories leading to observing run four. Galaxies.

[5] Nardecchia I. (2022). Detecting gravitational waves with Advanced Virgo. Galaxies.

[6] Arun K., Belgacem E., Benkel R., Bernard L., Berti E., Bertone G., Besancon M., Blas D., Böhmer C.G., Brito R. (2022). New horizons for fundamental physics with LISA. Living Rev. Relativ..

[7] Wang J., Qi K.Q., Wang S.X., Gao R.H., Li P., Yang R., Liu H.S., Luo Z.R. (2024). Advance and prospect in the study of laser interferometry technology for space gravitational wave detection. Sci. Sin.-Phys. Mech. Astron..

[8] Wang S., Liu D., Zhan X., Dong P., Shen J., Wang J., Gao R., Guo W., Xu P., Qi K. (2024). Core Payload of the Space Gravitational Wave Observatory: Inertial Sensor and Its Critical Technologies. Sensors.

[9] Zhang W., Lei J., Wang Z., Li C., Yang S., Min J., Wen X. (2024). Finite Element Analysis of Electrostatic Coupling in LISA Pathfinder Inertial Sensors. Sensors.

[10] Chen J., Liu C., Zhang Y.L., Wang G. (2025). Alternative LISA-TAIJI networks: Detectability of parity violation in stochastic gravitational wave background. Phys. Rev..

[11] Hartig M.S., Schuster S., Wanner G. (2022). Geometric tilt-to-length coupling in precision interferometry: Mechanisms and analytical descriptions. J. Opt..

[12] Wang Y., Meng L., Xu X., Niu Y., Qi K., Bian W., Yang Q., Liu H., Jia J., Wang J. (2021). Research on Semi-Physical Simulation Testing of Inter-Satellite Laser Interference in the China Taiji Space Gravitational Wave Detection Program. Appl. Sci..

[13] Wang Z., Yu T., Zhao Y., Luo Z., Sha W., Fang C., Wang Y., Wang S., Qi K., Wang Y. (2019). Research on Telescope TTL Coupling Noise in Intersatellite Laser Interferometry. Photonic Sens..

[14] Chwalla M., Danzmann K., Álvarez M.D., Delgado J.E., Fernández Barranco G., Fitzsimons E., Gerberding O., Heinzel G., Killow C.J., Lieser M. (2020). Optical Suppression of Tilt-to-Length Coupling in the LISA Long-Arm Interferometer. Phys. Rev. Appl..

[15] Schuster S., Tröbs M., Wanner G., Heinzel G. (2016). Experimental demonstration of reduced tilt-to-length coupling by a two-lens imaging system. Opt. Express.

[16] Yu M., Wu Y., Lin H.a., Huang L., Zou D., Liu Y., Li H., Li J., lin L. (2025). Tilt-to-length coupling noise suppression method for the manufacturing of space gravitational wave telescopes. Precis. Eng..

[17] Hartig M.S., Wanner G. (2023). Tilt-to-length coupling in LISA Pathfinder: Analytical modeling. Phys. Rev..

[18] Edwards P., Dave M., Weaver A., Zhao M., Fulda P., Mueller G., Wanner G. (2025). Impact of phase signal formulations on tilt-to-length coupling noise in the LISA test mass interferometer. Class. Quantum Gravity.

[19] Wang Z., Yang S., Jia F., Wu K., Liao F., Duan H., Yeh H.C. (2024). Alternative Approach to Tilt-to-Length Coupling Estimation for Laser Ranging Interferometers in Future Gravity Missions. Remote Sens..

[20] Chwalla M., Danzmann K., Barranco G.F., Fitzsimons E., Gerberding O., Heinzel G., Killow C.J., Lieser M., Perreur-Lloyd M., Robertson D.I. (2016). Design and construction of an optical test bed for LISA imaging systems and tilt-to-length coupling. Class. Quantum Gravity.

[21] Qian X.G., Cui Z., Shi H.Q., Wang X., Yao W.L., Gao R.H., Wang Y.K. (2024). Analysis of beam walk in inter-satellite laser link: Implications for Differential Wavefront Sensing in Gravitational Wave Detection. Appl. Sci..

[22] Li J.C., Lin H.A., Luo J.X., Wu Y.X., Wang Z. (2022). Optical design of space gravitational wave detection telescope. Chin. Opt..

[23] Xie F., Peng X., Tang W., Zhao M., Ma X. (2025). Multiparameter hierarchical sensitivity analysis of tilt-to-length coupling noise in Taiji science interferometer. Chin. Phys..

[24] Shaddock D., Ware B., Halverson P., Spero R., Klipstein B. (2006). Overview of the LISA Phasemeter. AIP Conf. Proc..

